# Mechanistic Insight into Yeast Bloom in a Lactic Acid Bacteria Relaying-Community in the Start of Sourdough Microbiota Evolution

**DOI:** 10.1128/Spectrum.00662-21

**Published:** 2021-10-20

**Authors:** Mugihito Oshiro, Masaru Tanaka, Rie Momoda, Takeshi Zendo, Jiro Nakayama

**Affiliations:** a Laboratory of Microbial Technology, Division of Systems Bioengineering, Department of Bioscience and Biotechnology, Faculty of Agriculture, Graduate School, Kyushu University, Fukuoka, Japan; b Central Laboratory of Yamazaki Baking Company Limited, Chiba, Japan; University of Melbourne

**Keywords:** lactic acid bacterial relay, natural food fermentation, metabolic interaction, microbial community evolution, *Saccharomyces cerevisiae*, bakery

## Abstract

The spontaneous microbiota of wheat sourdough, often comprising one yeast species and several lactic acid bacteria (LAB) species, evolves over repeated fermentation cycles, which bakers call backslopping. The final product quality largely depends on the microbiota functions, but these fluctuate sometimes during the initial months of fermentation cycles due to microbiota evolution in which three phases of LAB relay occur. In this study, the understanding of yeast-LAB interactions in the start of the evolution of the microbiota was deepened by exploring the timing and trigger interactions when sourdough yeast entered a preestablished LAB-relaying community. Monitoring of 32 cycles of evolution of 6 batches of spontaneous microbiota in wheat sourdoughs revealed that sourdough yeasts affected the LAB community when the 2nd- or 3rd-relaying types of LAB genera emerged. In *in vitro* pairwise cocultures, all 12 LAB strains containing the 3 LAB-relaying types arrested the growth of a Saccharomyces cerevisiae strain, a frequently found species in sourdoughs, to various extents by sugar-related interactions. These findings suggest competition due to different affinities of each LAB and a S. cerevisiae strain for each sugar. In particular, maltose was the driver of S. cerevisiae growth in all pairwise cocultures. The functional prediction of sugar metabolism in sourdough LAB communities showed a positive correlation between maltose degradation and the yeast population. Our results suggest that maltose-related interactions are key factors that enable yeasts to enter and then settle in the LAB-relaying community during the initial part of evolution of spontaneous sourdough microbiota.

**IMPORTANCE** Unpredictable evolution of spontaneous sourdough microbiota sometimes prevents bakers from making special-quality products because the unstable microbiota causes the product quality to fluctuate. Elucidation of the evolutionary mechanisms of the sourdough community, comprising yeast and lactic acid bacteria (LAB), is fundamental to control fermentation performance. This study investigated the mechanisms by which sourdough yeasts entered and settled in a bacterial community in which a three-phase relay of LAB occurred. Our results showed that all three layers of LAB restricted the cohabiting yeast population by competing for the sugar sources, particularly maltose. During the initial evolution of spontaneous sourdough microbiota, yeasts tended to grow synchronously with the progression of the lactic acid bacterial relay, which was predictably associated with changes in the maltose degradation functions in the bacterial community. Further study of ≥3 species’ interactions while considering yeast diversity can uncover additional interaction mechanisms driving the initial evolution of sourdough microbiota.

## INTRODUCTION

Spontaneous fermented sourdough is used to produce various foods (1–3). The quality of the foods largely depends on the fermentation by the sourdough microbiota, which often comprises one yeast species and several lactic acid bacteria (LAB) species (4, 5). To date, >30 yeast species and >70 LAB species have been identified in sourdoughs (4, 6). These diverse species combine to form various species combinations of sourdough microbiota.

Maltose, the most abundant sugar in wheat sourdough, is essential for sustaining wheat sourdough ecosystems (6). The maltose-fermenting yeast Saccharomyces cerevisiae has been reported as the most common yeast species in 68% of 394 sourdoughs (7). The outstanding ability of S. cerevisiae is its dough-leavening performance due to vigorous CO_2_ production (8). In addition, S. cerevisiae extracellularly converts sucrose into glucose and fructose by invertase activity (9).

The microbial activity of spontaneous sourdoughs in Europe and the United States has traditionally been maintained by repeating fermentation cycles, a practice that bakers call backslopping (5). Some bakeries in San Francisco have maintained sourdough by backslopping over 150 years (10). In Japan, spontaneous sourdoughs are sometimes discarded after several months of repeated fermentation cycles and replaced with newly prepared spontaneous sourdoughs. Throughout the repeated fermentation cycles, bakers attempt to prepare the desirable sourdough microbiota but are sometimes hampered by microbial community shifts, resulting in unstable product quality (11). Thus, a deeper understanding of the evolutionary mechanisms of spontaneous sourdough microbiota during the initial months is required. This information is fundamental in developing a sourdough microbial starter while considering complex microbial succession.

The evolution of spontaneous sourdough microbiota was initiated by allowing the pioneer species of LAB to grow within the first several fermentation cycles (11). Subsequently, the succession of the LAB community, referred to as the LAB relay, occurred (6). The LAB relay is explained by three groups of LAB genera: (i) *Enterococcus*, *Lactococcus*, *Leuconostoc*, and *Weissella*; (ii) *Pediococcus* and *Latilactobacillus*; and (iii) *Limosilactobacillus*, *Levilactobacillus*, *Lactiplantibacillus*, *Companilactobacillus*, *Fructilactobacillus*, and *Furfurilactobacillus*. However, according to our review of the literature, the time when a yeast enters the spontaneously relaying LAB community, and the trigger interactions between the yeast and LAB at that time, are unknown. Yeast-LAB interactions have been reported to be the force that maintains the stable microbiota of aged sourdoughs (4, 8).

16S rRNA gene amplicon sequencing is a powerful tool for observing the LAB community dynamics of sourdough (12). *In vitro* culture-based experiments provide mechanistic data that cannot be obtained using 16S rRNA amplicon sequencing (13). In several studies of fermented food microbiota, including sourdough, microbial interaction patterns were successfully verified by introducing a culture-based *in vitro* model that mimics the environment (14–17).

The aim of this study was to elucidate the key interactions that allow a yeast to enter the spontaneously relaying LAB community of sourdough throughout the evolution. Therefore, the temporal dynamics of the microorganisms and the predicted function of the bacteriota were investigated in 32 cycles of spontaneous wheat sourdough fermentation. In addition, the patterns and mechanisms of the interactions between yeast and LAB isolates were verified by *in vitro* culture-based experiments.

## RESULTS

### Time course monitoring of yeasts and LAB during sourdough microbiota evolution.

**FIG 1:**
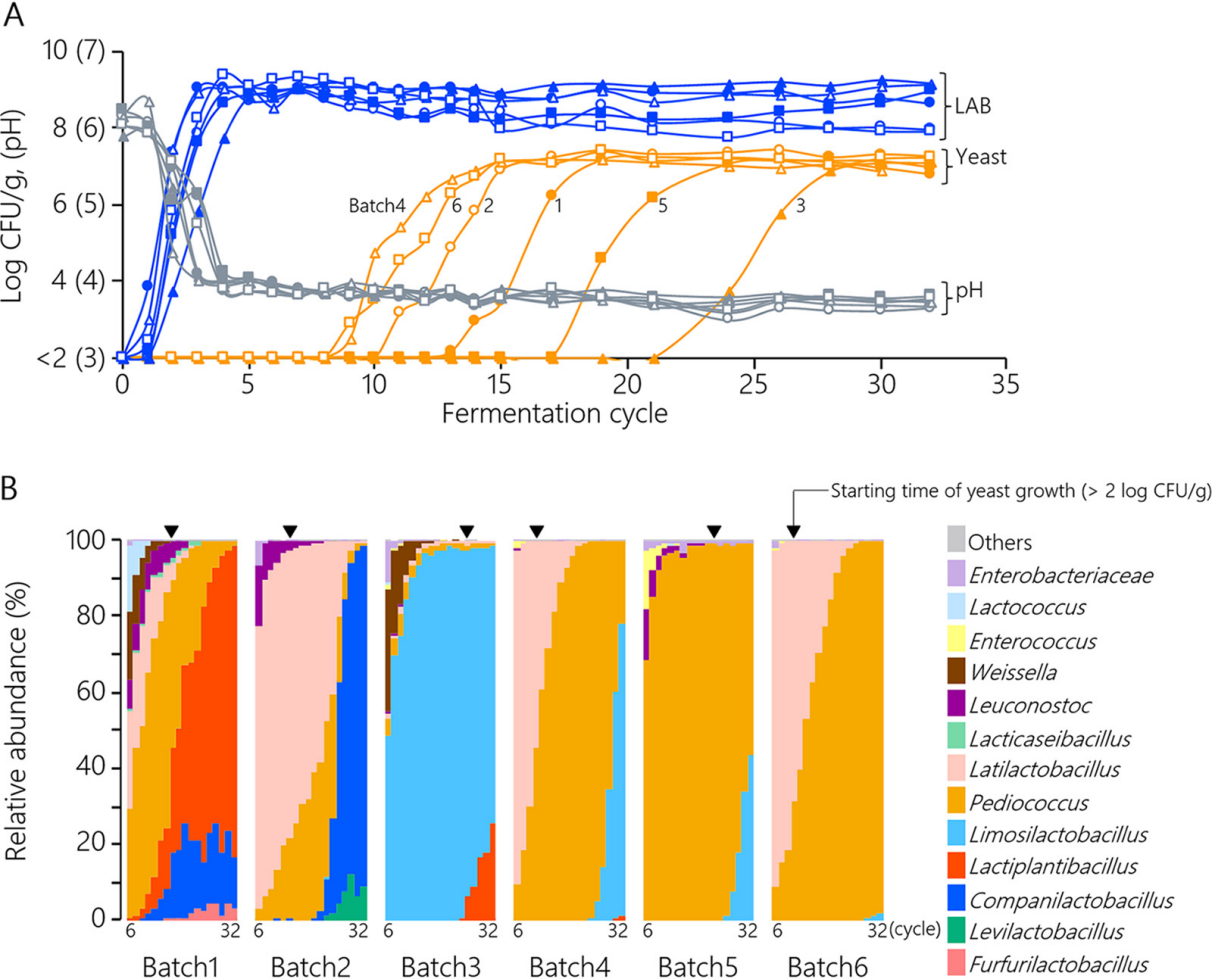
Microbial dynamics and pH during 32 fermentation cycles of 6 batches of wheat sourdough. (A) Changes in microbial population and pH. Closed circles, batch 1; open circles, batch 2; closed triangles, batch 3; open triangles, batch 4; closed squares, batch 5; open squares, batch 6. Gray lines, pH; blue lines, lactic acid bacteria; yellow lines, yeasts. Scale values of pH are shown in parentheses. (B) Bacterial community dynamics. Taxa present at ≤0.75% of the highest relative abundance for any sample were grouped as “others.”

**FIG 2:**
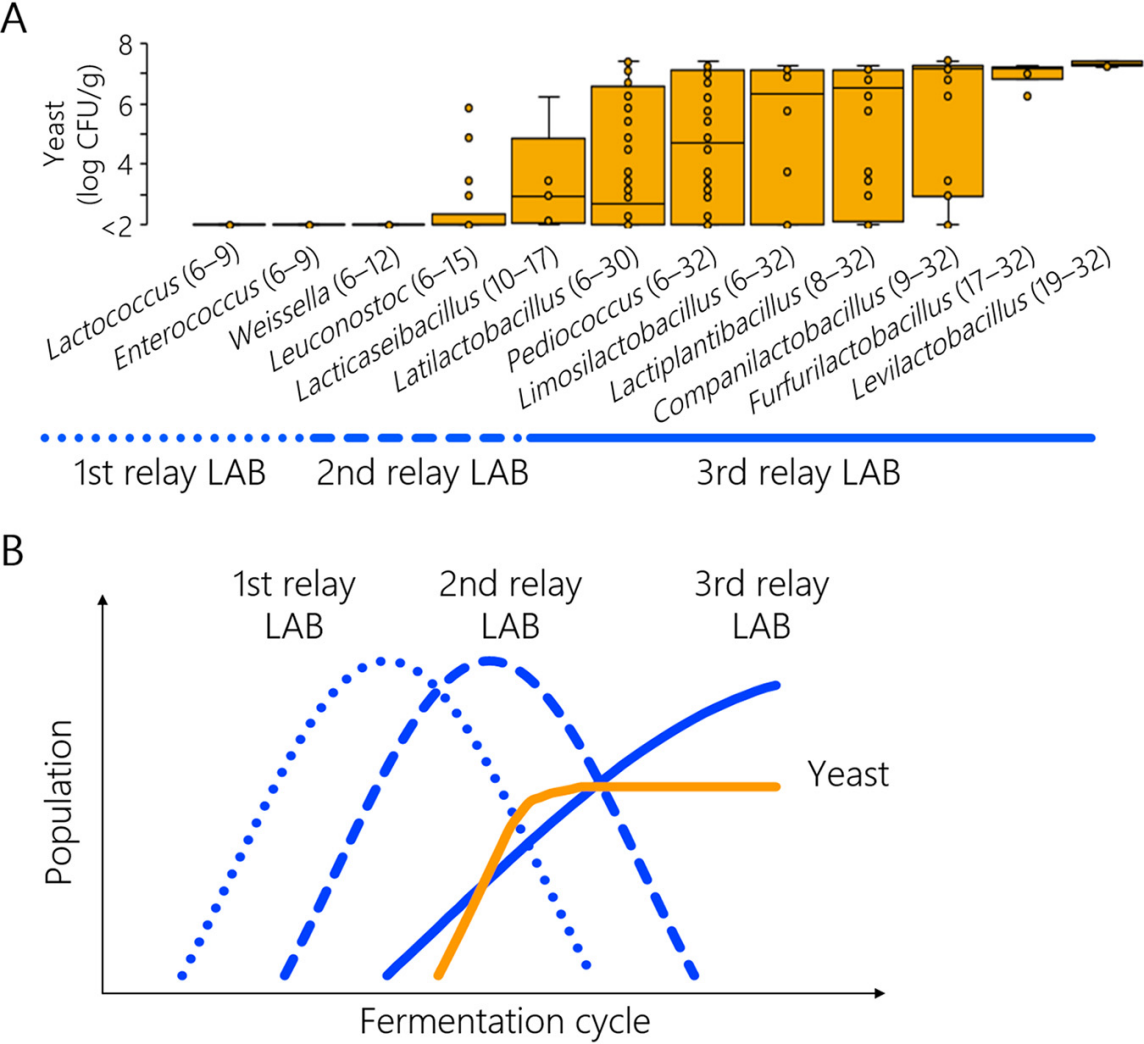
Relationship between sourdough lactic acid bacteria (LAB) relay and yeast population dynamics. (A) Succession of yeast population against changes in the LAB community. Yeast population is plotted when the indicated LAB genus constituted ≥1% of the total bacterial community. The corresponding fermentation cycles are shown in parentheses. (B) Proposed model of LAB and yeast relay during the sourdough fermentation process.

Microbial changes in six batches of spontaneous sourdoughs were monitored during 32 fermentation cycles in order to investigate diverse patterns of the microbial succession. After kneading wheat flour with tap water (cycle 0), all microbial counts were below the detection level (<2 log CFU/g), which indicated that the culturable cells of LAB or yeasts were low in the ingredients. During the first six cycles, the LAB of all sourdoughs increased to >8.6 log CFU/g while acidifying until the pH values were <4.0 (Fig. 1A). An increase in the yeast population was observed after an increase in LAB. Notably, the time at which the yeast population began to increase differed among batches, and the yeast counts of all sourdoughs reached 6.8 to 7.3 log CFU/g in the last cycle. 16S rRNA gene amplicon sequencing revealed that the bacteriota mainly comprised LAB and relayed continuously in all sourdoughs (Fig. 1B). Regardless of the differences in the LAB relay patterns among batches, the yeast population increased when the 2nd- and 3rd-relay LAB emerged during the fermentation cycles (Fig. 2). These results indicate that sourdough yeast interacted with several LAB genera in the community during the evolution of the microbiota.

### Isolation and identification of sourdough LAB and yeast.

Individual interaction patterns between a LAB strain and a yeast strain were verified by using 12 LAB strains, which were selected to cover all LAB relay types, in *in vitro* experiments (Table 1). The yeast was fixed with the S. cerevisiae 954-YBC-2021 strain; this strain was used because of its good fermentation performance in a spontaneous wheat sourdough (11). All strains were isolated from spontaneous sourdoughs or mixtures. Sanger sequencing revealed that the identities of all strains were ≥98.63% with type strains, except for the *pheS* gene of Furfurilactobacillus rossiae (90.89%).

**TABLE 1:** Sourdough isolates used for the *in vitro* experiments

Strain no.^*a*^	Identified species	LAB relay type	Identity (%) for:^*b*^			DDBJ accession no.	Isolation date (mo/day/yr)	Isolation source	Reference or source
			16S rRNA	*pheS*	26S rRNA				
1120-YBC-2021	Enterococcus durans	1st	100.00	98.72	NA	LC632336, LC632351	10/18/2017	Batch 5	This study
98-YBC-2021	Lactococcus lactis	1st	100.00	99.48	NA	LC632324, LC632339	4/26/2017	Spontaneous sourdough	11
35-YBC-2021	Leuconostoc citreum	1st	99.56	99.75	NA	LC632329, LC632344	4/25/2017	Spontaneous sourdough	11
2027-YBC-2021	Weissella cibaria	1st	99.80	98.70	NA	LC632325, LC632340	6/29/2018	Mixture of 152 spontaneous sourdoughs	This study
2008-YBC-2021	Weissella confusa	1st	100.00	99.48	NA	LC632332, LC632347	6/28/2018	Mixture of 152 spontaneous sourdoughs	14
118-YBC-2021	Latilactobacillus curvatus	2nd	99.68	99.57	NA	LC632330, LC632345	4/26/2017	Spontaneous sourdough	11
1938-YBC-2021	Pediococcus pentosaceus	2nd	99.64	99.74	NA	LC632326, LC632341	6/26/2018	Mixture of 152 spontaneous sourdoughs	This study
2206-YBC-2021	*Fructilactobacillus sanfranciscensis*	3rd	100.00	100.00	NA	LC632327, LC632342	7/5/2018	Mixture of 152 spontaneous sourdoughs	This study
2086-YBC-2021	*Furfurilactobacillus rossiae*	3rd	99.85	90.89	NA	LC632333, LC632348	7/1/2018	Mixture of 152 spontaneous sourdoughs	This study
504-YBC-2021	*Lactiplantibacillus plantarum*	3rd	99.83	100.00	NA	LC632335, LC632350	5/15/2017	Spontaneous sourdough	11
2092-YBC-2021	*Levilactobacillus brevis*	3rd	99.92	99.41	NA	LC632328, LC632343	7/1/2018	Mixture of 152 spontaneous sourdoughs	14
2211-YBC-2021	Limosilactobacillus fermentum	3rd	99.86	98.63	NA	LC632334, LC632349	7/5/2018	Mixture of 152 spontaneous sourdoughs	14
954-YBC-2021	Saccharomyces cerevisiae	NA	NA	NA	100.00	LC632338	6/23/2017	Spontaneous sourdough	11

a All strains were isolated from wheat sourdough prepared in our laboratory.

b NA, not applicable.

### *In vitro* examination of the effect of LAB genera on a yeast population.

**FIG 3:**
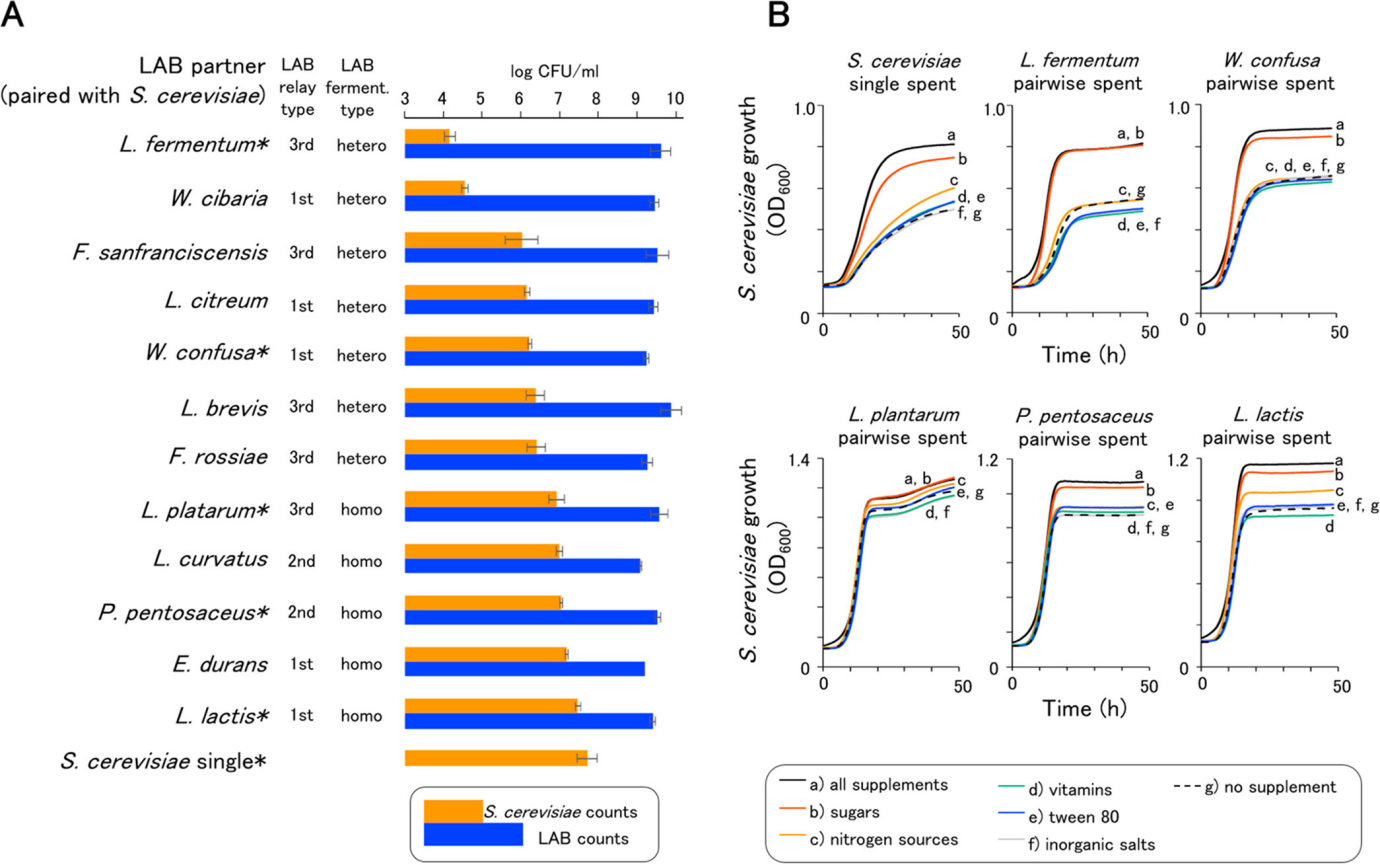
*In vitro* investigation of yeast-LAB interaction patterns. (A) The interaction patterns in pairwise coculture experiments. Microbial counts were measured at 14 fermentation cycles. S. cerevisiae was monococultured as a control. The coculture tests were performed in at least two batches, and the average values are shown with error bars (standard deviation). The asterisked coculture tests were used for the spent medium assays. (B) S. cerevisiae growth assay in spent medium supplemented with five nutrition groups from the medium. Reproducibility was confirmed using the same assay on another batch.

LAB and yeast populations were investigated using the 14-cycle repeated fermentation of the *in vitro* pairwise coculture. S. cerevisiae growth was lower in all pairwise cocultures with LAB than that in S. cerevisiae monococulture (Fig. 3A). Yeast population counts varied from 4.2 to 7.5 log CFU/ml, depending on the paired LAB strains. Heterofermentative LAB suppressed S. cerevisiae growth more than homofermentative LAB. LAB counts converged in the range of 9.1 to 9.9 log CFU/ml. The main factor determining the suppression of S. cerevisiae growth was identified using the spent media of several *in vitro* cocultures (Fig. 3B). The assays of S. cerevisiae inoculations into the spent media showed that supplementation with a sugar mixture improved S. cerevisiae growth.

### Sugar-utilizing characteristics of sourdough yeast and LAB strains.

**FIG 4:**
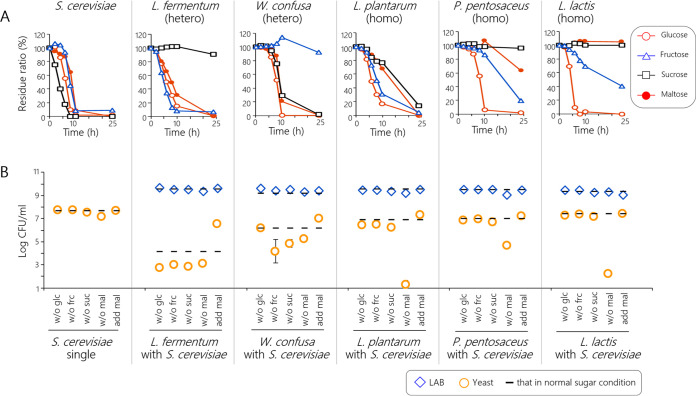
Sugar-utilizing properties of S. cerevisiae and LAB strains. (A) *In vitro* mixed-sugar utilization profile. The pure culture contained approximately 3 g/liter each of glucose, fructose, sucrose, and maltose. The lactic acid fermentation type is shown in parenthesis. The average values of two batch experiments are shown. Standard deviations are all below 6.6%. (B) *In vitro* pairwise coculture using the media with various sugar concentrations; w/o glc, no glucose; w/o frc, no fructose; w/o mal, no maltose; add mal, additional maltose. Microbial counts were measured at 14 fermentation cycles. The fermentation tests were performed in at least two batches, and the average values are shown with error bars (standard deviation).

The time course of mixed-sugar utilization was investigated using pure cultures. S. cerevisiae utilized all sugars almost simultaneously, with relatively high utilization rates (Fig. 4A). The mixed-sugar utilization patterns of LAB strains could be categorized by fermentation type. Two heterofermentative strains, namely, Limosilactobacillus fermentum and Weissella cibaria, utilized maltose regardless of the presence of other sugars (Table 2). Three homofermentative strains, Lactiplantibacillus plantarum, Pediococcus pentosaceus, and Lactococcus lactis, utilized maltose slowly and utilized glucose preferentially in the presence of mixed sugars. *L*. *fermentum*, the most negative interactor with S. cerevisiae found in this study, utilized all sugars, except for sucrose. However, because of the extracellular sucrose degradation by S. cerevisiae (see Fig. S1 in the supplemental material), the S. cerevisiae-*L. fermentum* pair was presumed to compete with other sugars. Furthermore, the innate disadvantages of S. cerevisiae on the growth rate, defined as *μ*_max_, and affinity of sugar, defined as *K_s_* (a lower *K_s_* represents a higher affinity), were greater than those of LAB (Table S1), possibly because the S. cerevisiae population tended to decrease when competing with LAB.

**TABLE 2:** Maximum sugar-utilizing rates of strains and S. cerevisiae population in the corresponding pairwise coculture

Strain	Maximum sugar-utilization rate (g/liter/h)				S. cerevisiae growth (log CFU/ml) in pairwise coculture^*a*^
	Glucose	Fructose	Sucrose	Maltose	
S. cerevisiae	22.8	24.0	17.7	27.8	Not applicable
*L. fermentum*	13.7	15.4	0.8	8.9	4.2
*W. confusa*	25.3	1.6	23.1	33.2	6.2
*L. plantarum*	16.1	11.2	6.1	6.0	6.9
P. pentosaceus	24.6	4.8	2.0	3.1	7.0
L. lactis	30.1	5.5	1.0	1.0	7.5

a The average population at 14 fermentation cycles, as shown in Fig. 3.

### Yeast-LAB pairwise cocultures using different sugar concentrations of media.

In *in vitro* pairwise cocultures using model media with different sugar-concentrations, variations in sugar composition influenced the cohabiting S. cerevisiae population (Fig. 4B). Removing maltose from the medium decreased the S. cerevisiae population to 1.3 to 5.2 log CFU/ml after the 14 fermentation cycles. Maltose removal in *L*. *plantarum* or L. lactis pairwise tests markedly decreased the S. cerevisiae population. This phenomenon probably occurred because S. cerevisiae preferentially utilized maltose in the cohabitation with either one of the two LAB strains. For similar reasons, fructose removal in the Weissella confusa pairwise test also decreased the S. cerevisiae population. Additionally, increasing the maltose concentration rescued the S. cerevisiae population to >6.5 log CFU/ml in all pairwise experiments, indicating that S. cerevisiae grew in response to the amount of maltose. Our results demonstrate that the influence of the patterns of LAB on S. cerevisiae was mainly determined by the maltose utilization abilities in all relay types of LAB harboring different sugar-utilizing characteristics.

### Sugar compositions and predictive functions of bacteriota in sourdough microbiota evolution.

**FIG 5:**
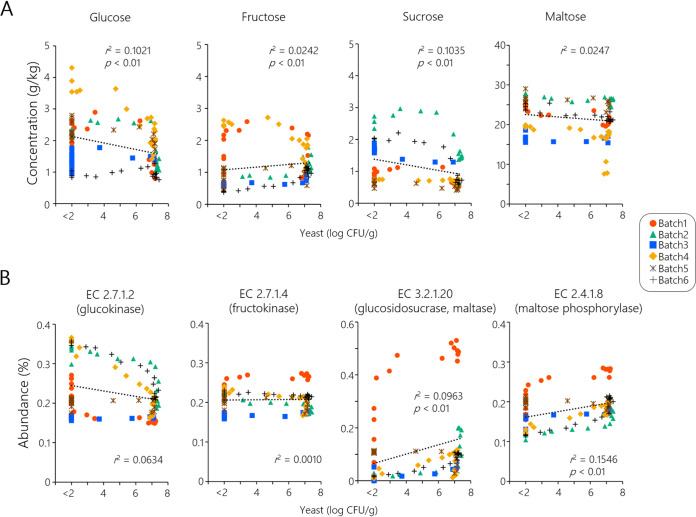
(A and B) Relationship between yeast populations in wheat sourdough and (A) sugar compositions or (B) sugar-associated functions of bacterial communities. The *P* value represents the significant difference between the sample group of high-yeast populations (≥7.0 log CFU/g) and that of low-yeast populations (≤2.0 log CFU/g) by Mann-Whitney *U* test. (A) Sugar concentrations against yeast populations. (B) Abundance of sugar-associated bacterial functions predicted by PICRUSt2 against yeast populations.

The relationship between the sugar-related factors and sourdough yeast bloom was investigated by analyzing the sugar compositions and predictive functions of bacteria in six batches of sourdough. Significant differences in glucose, fructose, and sucrose concentrations were detected between the sample groups of different yeast populations, and sugar levels were mostly maintained at constant concentrations throughout the fermentation cycles (Fig. 5A). Maltose was maintained at the highest concentration (average 22 g/kg), and the other sugars were maintained at low levels (average, 1 to 2 g/kg each). A phylogenetic investigation of communities by reconstruction of unobserved states (PICRUSt version 2) analysis was conducted using the 16S amplicon sequencing data (Fig. 5B). The pathway abundance of enzyme commission (EC) numbers was compared between the sample groups of yeast population levels. The results showed that disaccharide-degradation pathways, namely, glucosidosucrase and maltase (EC 3.2.1.20) and maltose phosphorylase (EC 2.4.1.8), were significantly enriched when the yeast population increased. As shown in Table S2, pathway abundances of alanine, aspartate, and glutamate metabolism were significantly enriched (single-asterisked EC numbers); on the other hand, those of phenylalanine, tyrosine, and tryptophan metabolism were significantly reduced (double-asterisked EC numbers).

## DISCUSSION

Studies have examined limited species combinations in sourdough yeast-LAB interactions and mainly focused on Fructilactobacillus sanfranciscensis interactions with either Kazachstania humilis, Kazachstania exigua, or S. cerevisiae (18–21). *F*. *sanfranciscensis* belongs to the last-relay type in the LAB community succession and is found in old-aged sourdoughs (22). In this study, yeast-LAB interactions considering LAB community sifts expanded the mechanistic understanding of the microbial community evolution. The main finding of this study was that maltose-related interactions are critical in the start of the evolution of sourdough microbiota in which three phases of LAB relay occurs. Maltose interactions could stabilize the yeast-LAB microbiota over 150 years (22, 23).

Different times of yeast outgrowth among six batches of sourdough fermentation raised an additional question. How much did the yeast inflow into the ecosystem influence the time of yeast outgrowth? Microbial entry routes into sourdough, such as flours, tap water, instruments, and the operator’s hands, have been reported (5, 24–26). One possibility is that a yeast with a low level (<10^2^ CFU/g) entered into spontaneous sourdough at approximately the same time as LAB by entering the aforementioned route and was ready and waiting for the progress of LAB relay.

Low rates of maltose utilization by homofermentative LAB probably maintain the cohabiting S. cerevisiae population because S. cerevisiae preferentially utilizes maltose. However, the patterns of LAB interaction with the yeast population in *in vitro* pairwise tests could presumably not be identified based on LAB relay types. This result implied that sourdough microbial communities concealed other interaction forces that could not be detected in *in vitro* pairwise tests (17). In an attempt to mimic sourdough communities more closely, high-order (≥3 species) interaction tests combining several LAB species and one yeast species will be conducted *in vitro* in further research. In conjunction with these tests, mathematical approaches using data-driven modeling and network analysis might facilitate the elucidation of high-order interaction mechanisms (27).

Maltose-nonfermenting yeasts have also been found in sourdough ecosystems (4, 28). This type of yeast shows different interactions with LAB from S. cerevisiae due to the avoidance of maltose competition with LAB (21). Interaction studies using maltose-nonfermenting yeasts will be necessary to identify additional interaction mechanisms underlying the start of evolution of sourdough yeast-LAB microbiota.

The correlation between the maltose degradation capacity of the LAB community and yeast growth suggests that the onset of yeast growth in sourdough is triggered by the increasing availability of maltose by maltose phosphorylase in the LAB community. Thus, what has been often exemplified is that maltose can be intracellularly converted into glucose and glucose 1-phosphate by maltose phosphorylase in sourdough LAB, and then glucose is released into the environment, where it becomes available for the cohabiting yeast (8, 29). A metatranscriptomic study of spontaneous sourdoughs also showed a slight increase in maltose phosphorylase expression level with fermentation cycles (30). Additionally, PICRUSt2 predictions in amino acid metabolism can reflect functional changes of the sourdough LAB community with yeast cohabitation.

In conclusion, the evolution of six batches of spontaneous microbiota showed that the yeast population increased upon entry into the second phase of three relaying phases of LAB community evolution. *In vitro* culture-based experiments showed that all of the three relaying types of LAB arrested the growth of an S. cerevisiae strain to different extents by sugar-related interactions, particularly those involving maltose, suggesting competition due to the different affinities of each LAB and yeast for each sugar. Functional prediction of sourdough LAB communities showed a positive correlation between maltose degradation and yeast population. These results indicate that maltose-related interactions are key factors for the entrance and settlement of sourdough yeasts into a preestablished LAB-relaying community during the initial part of the evolution process.

## MATERIALS AND METHODS

### Spontaneous wheat sourdough preparation.

**Fermentation cycle conditions.** Six batches of spontaneous wheat sourdough were prepared in our laboratory. Six bags of refined wheat flour were purchased from milling companies in Japan, and the same bag of flour was used for one batch of sourdough preparation. The ash content of wheat flour was 0.45% to 0.60%, and the protein content was 10.0% to 12.0%. The fermentation conditions and sampling protocols have been described previously (11). Briefly, 300 g of sourdough was fermented in a 1.5-liter plastic container for 60 days with 32 fermentation cycles. First, 150 g of wheat flour and tap water were mixed and fermented at 30°C for 8 h. Next, the mixture was stored at 4°C until the next cycle. At each cycle, 60 g of fermented sourdough was inoculated with a dough yield of 200. The sourdough was sampled at cycles 0 to 15, 17, 19, 21, 24, 26, 28, 30, and 32 of the fermentation. 16S rRNA gene amplicon sequencing and sugar analysis were performed using samples collected after six fermentation cycles. The pH was measured twice per sample, and the average values were obtained for each sample.

**16S rRNA gene amplicon sequencing.** Total genomic DNA was extracted from the sourdough by using the bead-beating method with the QIAamp DNA stool minikit (Qiagen, Hilden, Germany), and amplicon sequencing of 16S rRNA genes was performed using paired-end analysis (reagent v3 of the 600-cycle kit) with Illumina MiSeq system as previously described (11). The 16S rRNA V1-V2 region was amplified using the universal primers Tru27F and Tru354R (Table S3) (31).

The obtained sequences were processed with QIIME2 version 2019.7 (32) according to the tutorials. Initially, the imported paired-end sequences were demultiplexed. Amplicon sequence variants (ASVs) were obtained by performing quality filtering, denoising, chimera removal, and merging processes while removing the primer sequence using the DADA2 pipeline (33). The following parameters were used: trunc-len = 240 for forward reads and 220 for reverse reads, trim-left = 23 for forward reads and 20 for reverse reads, trunc-q = 10, and max-ee = 1. Taxonomy was determined using the naive Bayes classifiers trained to the 16S rRNA 27F/354R region by using the Silva database (Silva 132 99% operational taxonomic units [OTUs], full-length) (34). The reads assigned to chloroplasts or mitochondria were removed. A total of 2,903,554 reads from 277 ASVs were obtained from 108 samples (median, 28,500 reads per sample; range, 7,382 to 60,370 reads/sample). The relative abundance of each taxon was calculated as read counts/total read counts according to the ASV table. Prediction of metagenomic microbial functions was performed with PICRUSt2 (35) by using the standardized workflow with the ASVs from the DADA2 pipeline as input. Feature counts for calculating the core diversity metrics were rarified to 1,986,073. Excel 2016 (Microsoft Corporation) was used to draw the graphs.

**Statistics.** The significance of differences between the sample groups of different yeast populations was assessed; Mann-Whitney *U* tests were performed using wilcox.exact in R 4.0.2 (https://www.r-project.org) for each sugar concentration in the sourdoughs and for pathway abundance predicted by PICRUSt2.

### *In vitro* sourdough model experiments.

**Strains**. Enterococcus durans was isolated from spontaneous sourdough batch 5 in this study (Table 1). *W*. *cibaria*, P. pentosaceus, *F*. *sanfranciscensis*, and *F*. *rossiae* were isolated from the mixtures of 152 spontaneous sourdoughs, which were prepared according to a previous method (14). Seven LAB strains and one yeast strain of S. cerevisiae were previously isolated from spontaneous sourdoughs or mixtures by Oshiro et al. as reported in the literature (11, 14). All strains were isolated using the plate-culturing method (11). All isolates were genetically identified by Sanger sequencing. The primers used are shown in Table S3. The 16S rRNA gene of LAB was amplified using the primer pair 10F/800R (36, 37) or 27F/1492R (38). The *pheS* gene of LAB was amplified using pheS-21F/pheS-23-R primers (39). The D1/D2 domain of the 26S rRNA gene of yeast was amplified using NL1/NL4 primers (40). The amplified genes were directly sequenced by FASMAC Co., Ltd. (Kanagawa, Japan). The BLASTN (41) identities of all query sequences were derived from a comparison with the type strains. All NCBI accession numbers for the strains are listed in Table 1. The strains were frozen in 10% (wt/vol) glycerol at –80°C until use.

**Media.** Wheat sourdough simulation medium (WSSM) (42) was used for all *in vitro* experiments. WSSM mimicked the nutritional conditions of wheat sourdough, but this medium could not reproduce the continuous sugar generation from starch by flour amylases of sourdough. The formulation of WSSM changed only the sugar concentration in both the pure culture for the sugar-utilizing test and the coculture for additional pairwise tests. For the kinetic analysis of sugar-dependent growth, the nitrogen sources were replaced with 10 g/liter of Bacto peptone (Becton, Dickinson and Company, Franklin Lakes, NJ, USA) to reduce the import of unintended carbohydrate sources.

**Pairwise coculture experiments.** Each strain was individually precultivated in WSSM. Coculture was initiated by inoculating the two strains at a final optical density at 600 nm (OD_600_) of 0.01, in 1 ml of WSSM. The inoculation counts were 3.4 to 6.9 log CFU/ml for LAB and 4.4 to 5.2 log CFU/ml for S. cerevisiae. All cocultures reached ≥9.1 log CFU/ml for LAB and ≥5.8 log CFU/ml for S. cerevisiae within the six cycles. Next, CFU were counted after 14 cycles. For the control, a monococulture was inoculated with S. cerevisiae. The conditions of the fermentation cycle were the same as those for sourdough. The reproducibility was confirmed; each coculture experiment was performed in at least two batches.

Additional pairwise coculture experiments used WSSM containing different concentrations of glucose, fructose, sucrose, or maltose. One of the four sugars was removed from the medium, or 10 g/liter of maltose was added to the normal composition of WSSM. No other conditions were changed.

**Growth assays using spent medium.** The 16 cycles of the pairwise coculture medium (in the pairwise coculture experiment paragraphs, above) were used for preparing the spent medium. The spent medium was prepared by filtering the coculture medium through a 0.22-μm membrane filter (Millex-GV; Merck Millipore, Massachusetts, USA). The WSSM components were divided into five groups according to the nutritional following categories: sugars, nitrogen sources, vitamins, oleic acid, and inorganic salt. The detailed composition of each nutritional group is summarized in Table S4. The spent medium (180 μl) was supplemented with 20 μl of one of the nutritional groups in the WSSM. The final additive concentration of nutrition was one-10th that of the normal WSSM. In a flat-bottom 96-well microplate (AGC Techno Glass, Shizuoka, Japan), the growth assay was initiated by inoculating S. cerevisiae at a final OD_600_ of 0.01. Growth was monitored by measuring the OD_600_ every 15 min for 48 h using a Varioskan Lux instrument (Thermo Fisher Scientific) set at 30°C with 5 s of agitation every 5 min. Each assay was performed twice to confirm the reproducibility.

**Pure culture for profiling sugar-utilizing characteristics.** The pure cultured strain was inoculated at 10% (vol/vol) into 200 ml of WSSM containing 2.1 to 3.7 g/liter each of glucose, fructose, sucrose, and maltose. The initial microbial count was ≥10^6^ CFU/ml. The pure culture was statically cultivated at 30°C for 24 h in a 225-ml centrifuge tube (As One; Osaka, Japan). The maximum utilization rates for each sugar were determined using the same time-series data.

The sugar degradation activity present in the supernatant of S. cerevisiae culture medium was investigated by inoculating 10% (vol/vol) of the spent medium into 20 ml of WSSM containing 3 g/liter of glucose, fructose, sucrose, or maltose. Pure cultures and sugar degradation assays were performed with at least two batches to confirm the reproducibility.

**Kinetics of sugar-dependent growth of sourdough isolates.** The microbial growth kinetics was examined; the maximum specific growth rate (*μ*_max_) and substrate saturation constant (*K_s_*) were experimentally determined based on the Monod equation, {μ = *μ*_max_·[*S*]/(*K_s_* + [*S*])}, where *S* represents the sugar concentration (43). The experimental workflow is summarized in Fig. S2. In a 96-well microplate, the preculture of a strain at an OD_600_ of 0.01 was inoculated into 200 μl of the modified WSSM, which contained glucose, fructose, sucrose, or maltose in the range of 0 to 60 g/liter. Next, the OD_600_ was monitored every 15 min using a Varioskan Lux instrument until the stationary phase. Growth conditions were set at 30°C with 5 s of agitation every 5 min. This monitoring test was repeated in at least three batches to confirm the reproducibility.

### Microbial counting and sugar quantification.

Microbial counting of sourdoughs has been described previously (11). LAB and yeasts were counted after incubation on maltose MRS agar medium and potato dextrose agar medium, respectively. In the *in vitro* experiments, the drop-plate method (44) was used for microbial counting. The serially diluted cell suspensions were spotted at 2.5 μl × 8 spots (total, 20 μl) on the agar plate. The resultant colonies were counted using a plate with 5 to 80 colonies per 8 spots. Microbial counts were measured twice per sample, and the average values were obtained for each sample.

The concentrations of glucose, fructose, sucrose, and maltose were determined using a biosensor (BF-7; Oji Scientific Instrument, Osaka, Japan) (11). All sugar concentrations were measured twice per sample, and the average values were obtained for each sample.

### Data availability.

The raw data of 16S rRNA gene amplicon sequences were deposited in the DNA Data Bank of Japan (DDBJ; accession number DRA011910) and in GenBank under BioProject number PRJDB11555. Partial 16S or 26S rRNA gene sequences and *pheS* gene sequences of sourdough isolates were also deposited in DDBJ (see Table 1 for accession numbers).
