# Supplementary material for: Mechanistic Insight into Yeast Bloom in a Lactic Acid Bacteria Relaying-Community in the Start of Sourdough Microbiota Evolution
Source: Microbiol Spectr. 2021 Oct 20;9(2):e00662-21. doi: 10.1128/Spectrum.00662-21 (PMC8528097; doi:10.1128/Spectrum.00662-21)
Supplement: SUPPLEMENTAL FILE 1 — Supplemental material. Download SPECTRUM00662-21_Supp_1_seq8.pdf, PDF file, 0.8 MB [file spectrum00662-21_supp_1_seq8.pdf]

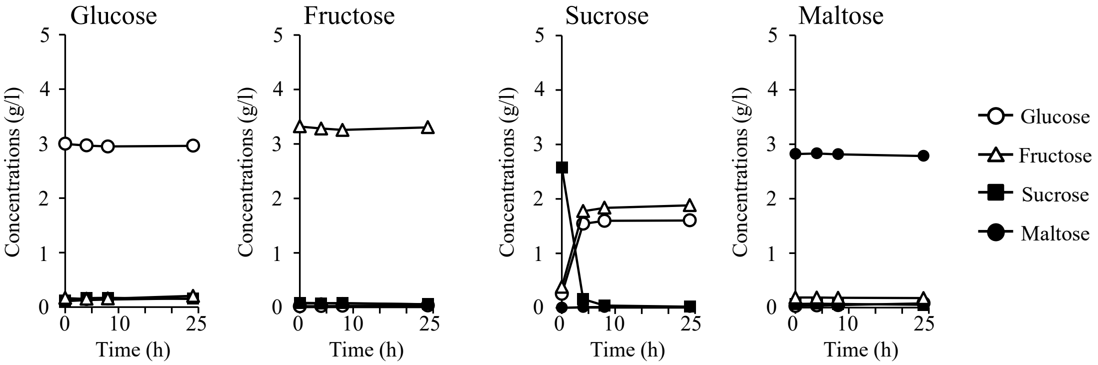

FIG S1 Extracellular sugar degradation activity profiles in the spent medium of *S. cerevisiae* mono-culture. Glucose, fructose, sucrose, or maltose was added to the spent medium. The average values of two batch experiments are shown. Standard deviations are all below 0.16 g/l.

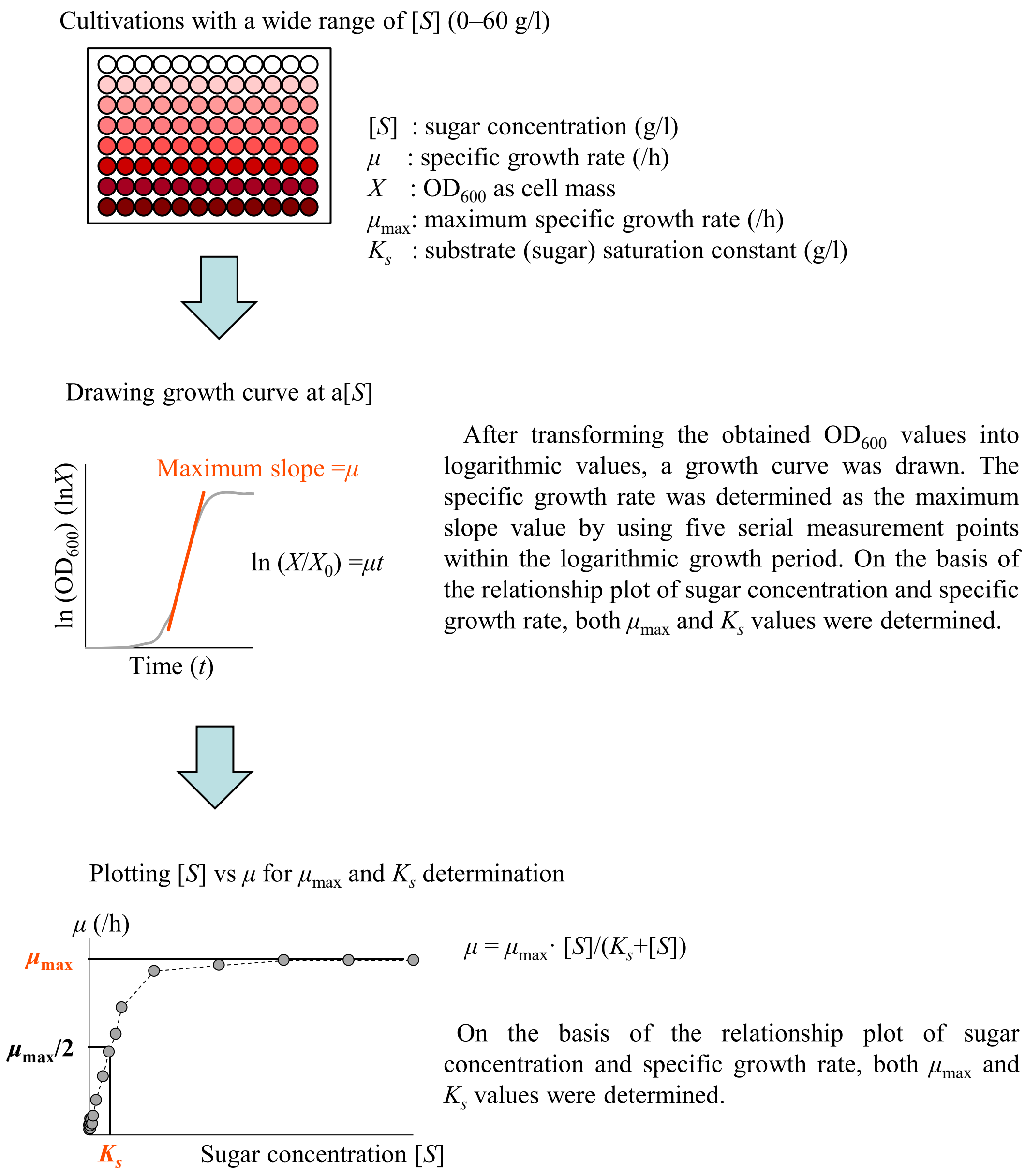

FIG S2 Workflow of kinetic growth characterization. μ_{max} and K_s were determined using the Monod equation. The values were determined against glucose, fructose, and maltose.

TABLE S1. Characteristics of kinetic growth of isolates [a]

Isolates	Glucose		Fructose		Maltose	
	μ_{max} (/h)	K_s (mg/l)	μ_{max} (/h)	K_s (mg/l)	μ_{max} (/h)	K_s (mg/l)
S. cerevisiae	0.43±0.01	575±222	0.36±0.04	316±103	0.27±0.01	376±72
W. confusa	0.47±0.13	77±9	0.19±0.05	182±62	0.45±0.01	73±4
L. plantarum	0.48±0.00	143±21	0.41±0.02	2105±369	0.46±0.04	91±15
P. pentosaceus	0.68±0.04	139±24	0.52±0.08	64±19	0.39±0.04	62±36
L. lactis	0.62±0.05	189±31	0.59±0.04	161±58	0.23±0.11	≥6000

[a] Kinetic characteristics are measured at least 3 times and the average value was represented with ± standard deviation.

TABLE S2. The top 40 enrichments or reductions of pathway abundances predicted by PICRUSt2 [a]

Top 40 enrichment		
Difference abundance	EC number	Function
5250	EC:2.7.1.69	GO:D-glucosamine PTS permease activity ; GO:0103111
4575	EC:2.3.1.79	GO:maltose O-acetyltransferase activity ; GO:0008925
3389	EC:4.2.1.53	GO:oleate hydratase activity ; GO:0050151
2504	EC:1.8.1.7	GO:glutathione-disulfide reductase (NADPH) activity ; GO:0004362
2491	EC:2.7.1.12	GO:gluconokinase activity ; GO:0046316
2313	EC:1.1.1.27	GO:L-lactate dehydrogenase activity ; GO:0004459
2244	EC:2.7.1.30	GO:glycerol kinase activity ; GO:0004370
2044	EC:2.7.6.5	GO:GTP diphosphokinase activity ; GO:0008728
2024	EC:5.4.2.12	phosphoglycerate mutase (not annotated by GO)
1978	EC:2.5.1.74	GO:1,4-dihydroxy-2-naphthoate octaprenyltransferase activity ; GO:0046428
1823	EC:2.7.1.56	GO:1-phosphofructokinase activity ; GO:0008662
1803	EC:3.6.3.44	xenobiotic-transporting ATPase (not annotated by GO)
1735	EC:3.6.3.40	teichoic-acid-transporting ATPase (not annotated by GO)
1729	EC:3.2.1.20	GO:alpha-1,4-glucosidase activity ; GO:0004558
1690	EC:3.5.1.25	GO:N-acetylglucosamine-6-phosphate deacetylase activity ; GO:0008448
1588	EC:2.3.1.117	GO:2,3,4,5-tetrahydropyridine-2,6-dicarboxylate N-succinyltransferase activity ; GO:0008666
1251	EC:3.4.21.102	GO:serine-type endopeptidase activity ; GO:0004252
1144	EC:5.3.1.6	GO:ribose-5-phosphate isomerase activity ; GO:0004751
1128	EC:1.3.1.9	GO:enoyl-[acyl-carrier-protein] reductase (NADH) activity ; GO:0004318, GO:0016631
1073	EC:6.3.5.5*	GO:carbamoyl-phosphate synthase (glutamine-hydrolyzing) activity ; GO:0004088
1051	EC:4.1.1.98	GO:3-octaprenyl-4-hydroxybenzoate carboxy-lyase activity ; GO:0008694
1007	EC:3.1.3.48	GO:protein tyrosine phosphatase activity ; GO:0004725
1002	EC:3.6.3.27	phosphate-transporting ATPase (not annotated by GO)
979	EC:5.4.2.6	GO:beta-phosphoglucomutase activity ; GO:0008801
958	EC:4.1.2.40	GO:tagatose-bisphosphate aldolase activity ; GO:0009025
946	EC:1.4.1.1*	GO:alanine dehydrogenase activity ; GO:0000286
939	EC:6.3.5.3	GO:phosphoribosylformylglycinamidine synthase activity ; GO:0004642
876	EC:2.7.1.31	GO:glycerate kinase activity ; GO:0008887
831	EC:1.2.1.20	GO:glutarate-semialdehyde dehydrogenase (NAD+) activity ; GO:0047949
827	EC:2.7.8.20	GO:phosphatidylglycerol-membrane-oligosaccharide glycerophosphotransferase activity ; GO:0008960
822	EC:6.1.1.13*	D-alanine-membrane acceptor ligase (not annotated by GO)
815	EC:1.2.1.79*	GO:succinate-semialdehyde dehydrogenase (NADP+) activity ; GO:0036243
808	EC:5.1.3.14	GO:UDP-N-acetylglucosamine 2-epimerase activity ; GO:0008761
786	EC:2.7.6.1	GO:ribose phosphate diphosphokinase activity ; GO:0004749
737	EC:1.2.1.16*	GO:succinate-semialdehyde dehydrogenase [NAD(P)+] activity ; GO:0009013
694	EC:1.2.1.11	GO:aspartate-semialdehyde dehydrogenase activity ; GO:0004073
692	EC:2.3.1.183	GO:phosphinothricin N-acetyltransferase activity ; GO:0102971
691	EC:2.4.1.8	GO:maltose phosphorylase activity ; GO:0050082
677	EC:2.3.1.128	GO:ribosomal-protein-alanine N-acetyltransferase complex ; GO:0009323
669	EC:3.4.11.5	GO:aminopeptidase activity ; GO:0004177
Top 40 reduction		
-3065	EC:1.1.1.1	GO:alcohol dehydrogenase (NAD+) activity ; GO:0004022
-2736	EC:1.3.98.1	GO:dihydroorotate dehydrogenase (fumarate) activity ; GO:1990663
-2416	EC:2.3.2.3	GO:lysyltransferase activity ; GO:0050071
-2393	EC:2.7.1.71**	GO:shikimate kinase activity ; GO:0004765
-2348	EC:3.2.1.23	GO:beta-galactosidase activity ; GO:0004565
-2300	EC:2.7.1.35	GO:pyridoxal kinase activity ; GO:0008478
-2038	EC:3.6.3.54	P-type Cu+ transporte (not annotated by GO)
-1990	EC:1.1.1.95	GO:phosphoglycerate dehydrogenase activity ; GO:0004617
-1976	EC:4.2.1.46	GO:dTDP-glucose 4,6-dehydratase activity ; GO:0008460
-1565	EC:3.5.4.16	GO:GTP cyclohydrolase I activity ; GO:0003934
-1477	EC:3.5.1.1	GO:asparaginase activity ; GO:0004067
-1406	EC:4.2.1.51**	GO:prephenate dehydratase activity ; GO:0004664
-1404	EC:6.3.2.17	GO:tetrahydrofolylpolyglutamate synthase activity ; GO:0004326
-1359	EC:6.3.2.12	GO:dihydrofolate synthase activity ; GO:0008841
-1357	EC:3.6.3.34	ABC-type ferric hydroxamate transporter (not annotated by GO)
-1251	EC:3.6.3.3	Cd2+-exporting ATPase (not annotated by GO)
-1164	EC:3.6.3.5	P-type Zn2+ transporter (not annotated by GO)
-1080	EC:5.99.1.2	DNA topoisomerase (not annotated by GO)
-1064	EC:1.8.1.9	GO:thioredoxin-disulfide reductase activity ; GO:0004791
-908	EC:3.4.11.9	GO:aminopeptidase activity ; GO:0004177
-900	EC:3.6.3.17	Acting on acid anhydrides (not annotated by GO)
-842	EC:4.1.2.4	GO:deoxyribose-phosphate aldolase activity ; GO:0004139
-830	EC:2.1.1.72	GO:site-specific DNA-methyltransferase (adenine-specific) activity ; GO:0009007
-819	EC:2.5.1.54**	GO:3-deoxy-7-phosphoheptulonate synthase activity ; GO:0003849
-784	EC:2.7.1.148	GO:4-(cytidine 5'-diphospho)-2-C-methyl-D-erythritol kinase activity ; GO:0050515
-708	EC:3.8.1.2	GO:(S)-2-haloacid dehalogenase activity ; GO:0018784

-705	EC:3.6.3.31	ABC-type polyamine transporter (not annotated by GO)
-679	EC:2.4.1.5	GO:dextransucrase activity ; GO:0047849
-672	EC:3.6.3.32	ABC-type quaternary amine transporter (not annotated by GO)
-634	EC:1.2.1.10	GO:acetaldehyde dehydrogenase (acetylating) activity ; GO:0008774
-620	EC:3.5.4.2	GO:pyrithiamine deaminase activity ; GO:0050239
-615	EC:2.7.7.9	GO:UTP:glucose-1-phosphate uridylyltransferase activity ; GO:0003983
-612	EC:3.4.22.70	sortase A (not annotated by GO)
-610	EC:2.7.1.45	GO:2-dehydro-3-deoxygluconokinase activity ; GO:0008673
-586	EC:3.1.3.25	GO:inositol monophosphate phosphatase activity ; GO:0052834
-573	EC:3.6.1.55	GO:8-oxo-7,8-dihydrodeoxyguanosine triphosphate pyrophosphatase activity ; GO:0035539
-565	EC:2.3.1.54	GO:formate C-acetyltransferase activity ; GO:0008861
-565	EC:4.2.1.10**	GO:3-dehydroquinate dehydratase activity ; GO:0003855
-561	EC:6.3.4.19	tRNAIle-lysidine synthase (not annotated by GO)
-539	EC:1.1.1.25	GO:shikimate 3-dehydrogenase (NADP+) activity ; GO:0004764

[a] The top 40 enrichments (reductions) of pathway abundances represent the significantly enriched (reduced) pathways with $p < 0.05$ in the sample group of high yeast-populations ($\geq$ 7.0 log CFU/g) compared to that of low yeast-populations ($\leq$ 2.0 log CFU/g) by Mann–Whitney *U* test. The difference abundance was calculated by subtracting the median value of the pathway abundances in the sample group of low yeast-populations from that of high yeast-populations. The function is mainly annotated by Gene Ontology (GO) mapping. Alanine, aspartate and glutamate metabolisms are indicated by single asterisk. Phenylalanine, tyrosine and tryptophan metabolisms are indicated by double asterisks.

TABLE S3. List of primers used in this study and their sequence

Name of primers	Primer sequence (5’–3’)	Target gene	Applied method
Tru27F	CGCTCTTCCGATCTCTGAGRGTTTGATYMTGGCTCAG	16S rRNA gene (V1–V2)	MiSeq 16S amplicon sequencing
Tru354R	TGCTCTTCCGATCTGACCTGCCTCCCGTAGGAGT		
10F	GTTTGATCCTGGCTCA	16S rRNA gene (V1–V4)	Sequencing for LAB isolates identification
800R	TACCAGGGTATCTAATCC		
27F	AGAGTTTGATCCTGGCTCAG	16S rRNA gene (V1–V9)	
1492R	GGTTACCTTGTTACGACTT		
pheS-21-F	CAYCCNGCHCGYGAYATGC	*pheS* gene	Sequencing for LAB species confirmation
pheS-23-R	GGRTGRACCATVCCNGCHCC		
NL1	GCATATCAATAAGCGGAGGAAAAG	26S rRNA gene (D1/D2)	Sequencing for yeast isolate identification
NL4	GGTCCGTGTTTCAAGACGG		

TABLE S4. Five categories of wheat sourdough simulation medium (WSSM) components

Nutrients	Concentration
Category: sugars	
Maltose	10 g/l
Sucrose	2 g/l
Glucose	0.5 g/l
Fructose	0.5 g/l
Category: nitrogen sources	
Wheat peptone	12 g/l
Granulated yeast extract	12 g/l
Category: vitamins	
Cobalamin	0.2 mg/l
Folic acid	0.2 mg/l
Nicotinamide	0.2 mg/l
Pantothenic acid	0.2 mg/l
Pyridoxal phosphate	0.2 mg/l
Thiamine	0.2 mg/l
Category: oleic acid	
Tween 80	1 g/l
Category: inorganic salt	
KH_2PO_4	4 g/l
K_2HPO_4	4 g/l
$MgSO_4$	0.1 g/l
$MnSO_4 \cdot H_2O$	0.1 g/l
